# The effect of anatomical variation on learning and cognitive load during simulation-based training of mastoidectomy—a randomized, controlled trial

**DOI:** 10.1007/s00405-026-10546-9

**Published:** 2026-08-20

**Authors:** Martin Frendø, Karoline Abildsø Arnesen, Mads Sølvsten Sørensen, Steven Arild Wuyts Andersen, Andreas Frithioff

**Affiliations:** 1 Department of Otorhinolaryngology—Head & Neck Surgery and Audiology, Copenhagen Hearing and Balance Center, Rigshospitalet, Blegdamsvej 9, Copenhagen, DK-2100 Denmark; 2 Center for HR & Education, Copenhagen Academy for Medical Education and Simulation (CAMES), Region H, Copenhagen, Denmark; 3 Department of Plastic and Breast Surgery, Zealand University Hospital, Roskilde, Denmark; 4 Department of Clinical Medicine, Faculty of Health and Medical Sciences, University of Copenhagen, Copenhagen, Denmark

**Keywords:** Mastoidectomy, Simulation based training, Virtual reality, Learning curve, Anatomical variation, Randomized controlled trial

## Abstract

**Purpose:**

To investigate the impact of anatomical variation (AV) on surgical skills acquisition, i.e., learning curves, and cognitive load (CL) during virtual reality (VR) simulation-based training (SBT) of mastoidectomy.

**Methods:**

A randomized, controlled educational trial. Trainees (medical students) were randomized to perform 12 virtual mastoidectomies on anatomically different temporal bones (intervention) or on the same temporal bone (controls). Training was organized as distributed practice. Mastoidectomy performances were evaluated by two blinded raters using the modified 26-points Welling Scale assessment tool and learning curves were established. CL was estimated by secondary task reaction time.

**Results:**

The intervention group (AV) improved from baseline performance by 80% compared with 59% in the control group (*p* = 0.52). The intervention group reached 68%, and the control group of 65% of the maximum performance score. The groups had equal CL at the beginning of training (49% increase in reaction time for the intervention group vs. 47% for the control group). After the training program, CL had decreased by 11.6% and 10% from baseline for the intervention and control group, respectively.

**Conclusion:**

Anatomical variation in SBT of mastoidectomy neither affected learning curves nor CL of novices compared to repeated practice on the same case. This suggests that AV is not needed for basic skills acquisition in complete novices. Nonetheless, AV and exposure to different anatomical variations could be valuable for more experienced trainees while other instructional designs might be more suitable for novice learners.

## Introduction

Simulation-based training (SBT) has become a cornerstone in surgical education, enabling trainees acquire fundamental surgical skills in a controlled environment [1]. By allowing for repeated practice, SBT can facilitate improvement of both technical and decision-making skills before progression to supervised training in the operating room [2, 3]. However, a disadvantage of SBT is that simulation often relies on a single standardized model, meaning that trainees are not exposed to varying anatomy. This lack of diversity could be problematic for several reasons: first, it contrasts real-life surgery, where adaptation to anatomical variation is essential. Second, training on the same model makes learning circumstances constant and predictable [4]. Finally, training on the same anatomical case does little to build the decision-making skills required for dealing with different patients’ anatomy [3].

To address these shortcomings, anatomical variation (AV) has been proposed as a potentially powerful instructional design feature in SBT [1, 5] AV likely represents a *desirable difficulty* by facilitating an active process of interpretation, often reported to improve retention of skills [4, 6].

Nonetheless, little is known about the effects of AV during surgical SBT, and few experimental studies have sought to elucidate how AV affects initial skills acquisition [5]. Recent work has begun to characterize the relationship between anatomical case difficulty and trainee performance in virtual reality mastoidectomy training; notably, expert-judged difficulty has not consistently predicted trainees actual performance [7], suggesting that the relationship between AV, case difficulty and skills development remains incompletely understood. Similarly, a randomized trial investigating the effect of introducing AV to CI training found that AV improved performance on selected subitems, but not on overall performance [8].

Learning curves are widely used in the medical education literature to represent trainees´ progression towards competence, including rate of learning and the point at which further practice yields diminishing progression (i.e. learning plateau) [9, 10]. In the specific context of mastoidectomy, numerous studies have consistently demonstrated a learning plateau where trainees often stagnate around the ninth simulated procedure, despite continued practice [11, 12]. This highlights the need to identify instructional interventions to break this plateau to elevate trainees’ skill level before progression to the OR. Anatomical variation represents a possible solution to this challenge.

Cognitive Load (CL) theory represents a cognitive approach to learning and describes how cognitive capacity is limited [13, 14] CL comprises different elements that can reduce or increase the learning capacity, being the task performed (intrinsic load), structures surrounding the task (extrinsic load) and the learning process (germane load) [13–16]. Consequently, the instructional design should balance the elements encompassed in CL, to allow sufficient excess cognitive capacity for formation of mental schemata that leads to acquisition and retention of skills [13].

AV might induce a higher CL, positively impacting learning due to increased cognitive engagement and improved formation of mental schemata. Nevertheless, AV could also induce a cognitive overload, where the stimuli during training exceed the trainee’s capacity, negatively impacting learning [17].

In this randomized controlled trial, we aimed to explore the effects of AV on skills acquisition (i.e., learning curves) and CL in novices learning the mastoidectomy procedure. We hypothesized that AV would slow initial learning and induce a higher CL but allow for improved skills acquisition and consolidation-.

## Materials and methods

### Study design

This was a randomized, controlled trial of an educational intervention (AV).

This study was part of a larger, multi-outcome randomized controlled trial, where we also explored the effects of AV on retention and transfer of skills [18].

### Participants and setting

Participants were medical students recruited from pre-clinical and clinical semesters at the University of Copenhagen. Recruitment was through social media, and the only exclusion criterion was previous VR mastoidectomy training. The study took place at CAMES, Rigshospitalet February–April 2021.

### Simulation platform

The Visible Ear Simulator (VES) is a VR temporal bone surgical simulator consisting of an academic freeware software program running on a standard gaming laptop ( https://visibleearsimulator.com). A haptic device (Geomagic Touch, 3D Systems, Rockhill, SC, USA) provides drilling with force feedback [19, 20].

There are seven different temporal bone cases available in VES version 3.5, allowing for training with anatomical variation [21]. Each case is also featured in both a left and right-sided version. The virtual patient cases are based on high-resolution cone-beam CT scans and the degree of anatomical variation reflects that of a normal patient population [22]. Consequently, major variations such as cochleovestibular malformations are not represented.

## Intervention

**Fig. 1:**
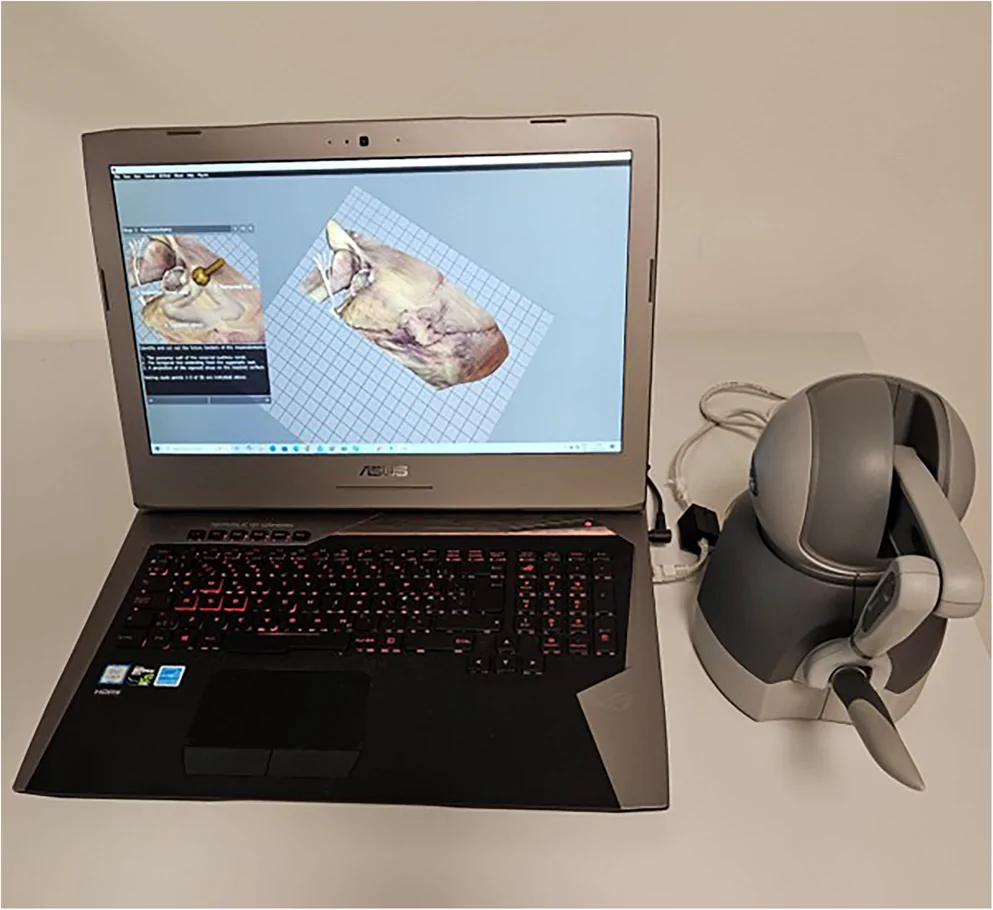
Setup of the computer running the visible ear simulator and the geomagic touch haptic device

First, participants completed a background questionnaire upon enrollment [18]. They were then introduced to the VR simulator (Fig. 1) and were allowed a 10-minute warm-up session to familiarize themselves with the equipment. The training program consisted of 12 mastoidectomy procedures (anatomical mastoidectomy and posterior tympanotomy) distributed over four training blocks spaced by 4–12 days [18]. As their only guidance during training, participants had access to an on-screen step-by-step dissection guide. The guide provided instructions on each step of the mastoidectomy procedure including images of the completed steps, serving as an in-built tutor. Each procedure had a time limit of 45 min.

The intervention group (AV) was given different cases for every procedure, including variation in laterality. The control group repeatedly trained on a single case. At the end of the training program, both the intervention and control group completed two procedures on two new anatomies which they had not been exposed to earlier. This served as an end-of-training test ensuring that comparison was not influenced by case familiarity or anatomical difficulty.

### Randomization

The participants were allocated into two groups using computer-generated randomization [23]. The two groups were randomized in a 1:1 ratio. In case of dropout, a new participant would be recruited to take their place.

### Sample size

Sample size calculations were based on previous studies [16, 24, 25]. We estimated that 12 participants in each group allows detection of a difference of 2.6 point (i.e., 10% of the modified Welling Scale) [18].

## Outcomes

The primary outcome was the learning curve, based on the performance throughout training. The 26-item modified Welling Scale was used to evaluate the final-product performance [26]. The assessment tool features 26 items rated binarily, giving a total score from zero to 26 points and is supported by extensive validity evidence [27]. Each final-product performance was assessed by two experts (co-authors XX and XX), who were blinded to participant and procedure data.

The secondary outcome was cognitive load (CL), estimated using secondary task reaction time—a quantitative method for CL estimation [28]. The relative reaction time is used as a proxy for the CL, indicating the participants relative use of mental capacity on the primary task [28]. Participants were exposed to an auditory stimulus prompting them to push a pedal during SBT. The time they used to perform this task was then measured and calculated as a percentage of the mean baseline duration for each training block (i.e., reaction time when not performing mastoidectomy training) [16]. We used a commercially available reaction timer (American Educational Products LLC, USA).

## Statistical methods

Linear mixed models (LMM) were used to analyze the final product performance scores due to repeated measurements [28]. The LMM model was built following the principles by Leppink et al. [29].

The final LMM included group, procedure and rater as fixed factors and a random intercept and random slope for procedure, nested within participant, were included to account for individual differences in baseline performance and learning rate. Interaction between group and procedure were tested to test whether the rate of skill acquisition (i.e. the slope of the learning curve) differed between the two groups but was not found to be significant (*P* = 0.525). Group was retained as fixed factor in the final model even though it did not reach statistical significance as it constitutes the primary exposure of interest. To characterize and illustrate the learning curves a secondary, descriptive LMM was fitted comparing baseline procedure and end-of-training. A comparable LMM approach, with group, procedure, and rater as fixed effects and a random intercept for participant, was used to analyze mean relative reaction times between groups. Estimated marginal means are reported. P-values < 0.05 were considered statistically significant. LMM was also used to analyze the mean relative reaction times between the two groups. SPSS version 27 for Mac (IBM, Chicago, IL, USA) was used for the analyses.

### Reporting

The reporting in this paper is based on the Consolidated Standards of Reporting Trials (CONSORT) guidelines extension for medical education research [30]. Statistical reporting is based on the SAMPL statistical reporting guidelines [31].

### Ethics

This trial was deemed exempted by the Ethics Committee of (H-21009521). Participants received no compensation for participation. Medical students were recruited to represent true novices since they have no exposure to the procedure during their pre-graduate training. The participation was voluntary and outside of school curriculum.

## Results

**Fig. 2:**
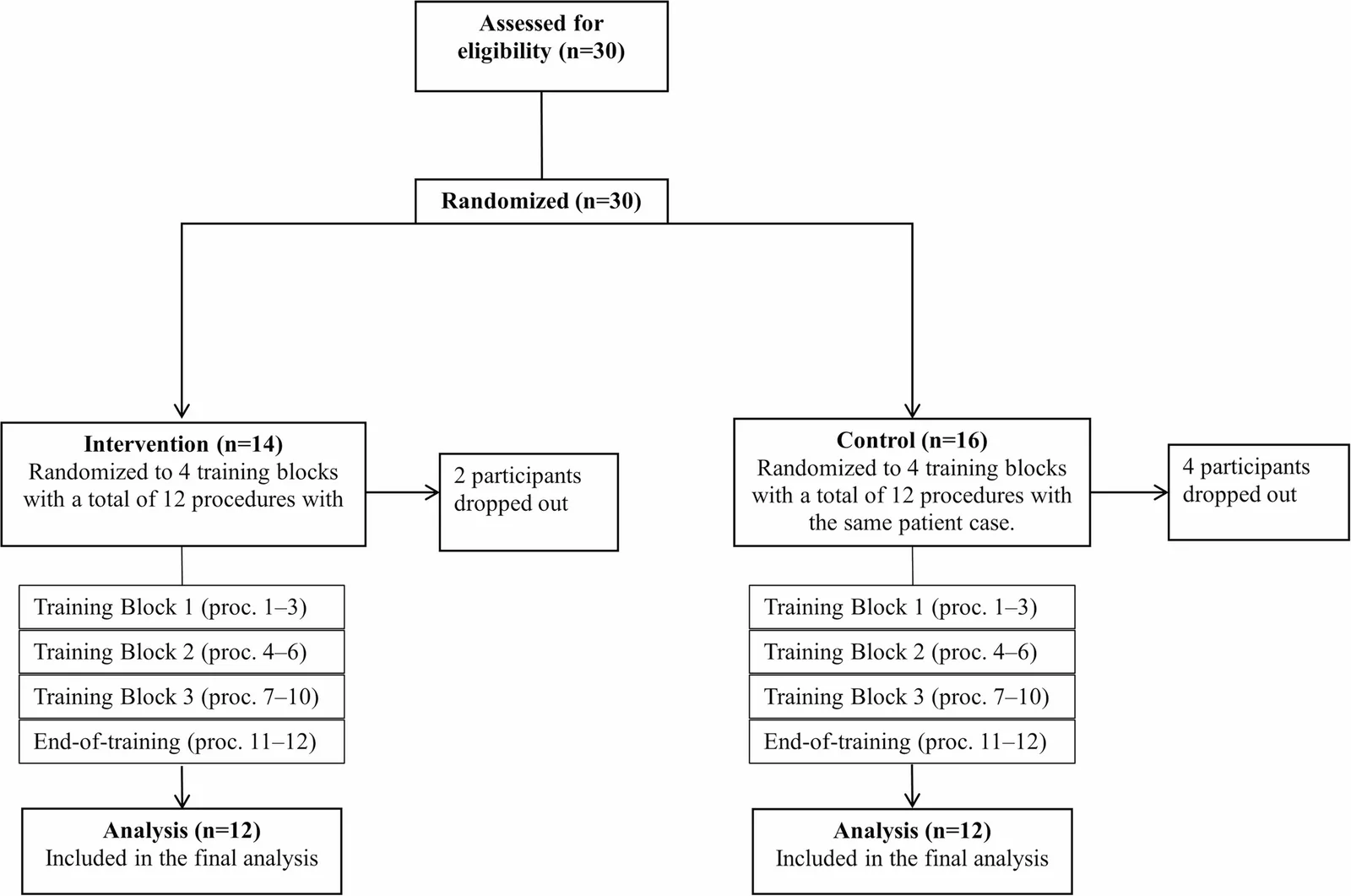
Study flowchart

A total of 30 participants were initially enrolled. Six dropped out during the study, evenly distributed between the two groups, citing time constraints as reason. Consequently, twenty-four participants were included: 12 in each group (Fig. 2). Twelve performances per participant were rated and included for analysis; one was missing for technical reasons, leaving 287 performance ratings for data analysis.

### Performance

**Fig. 3:**
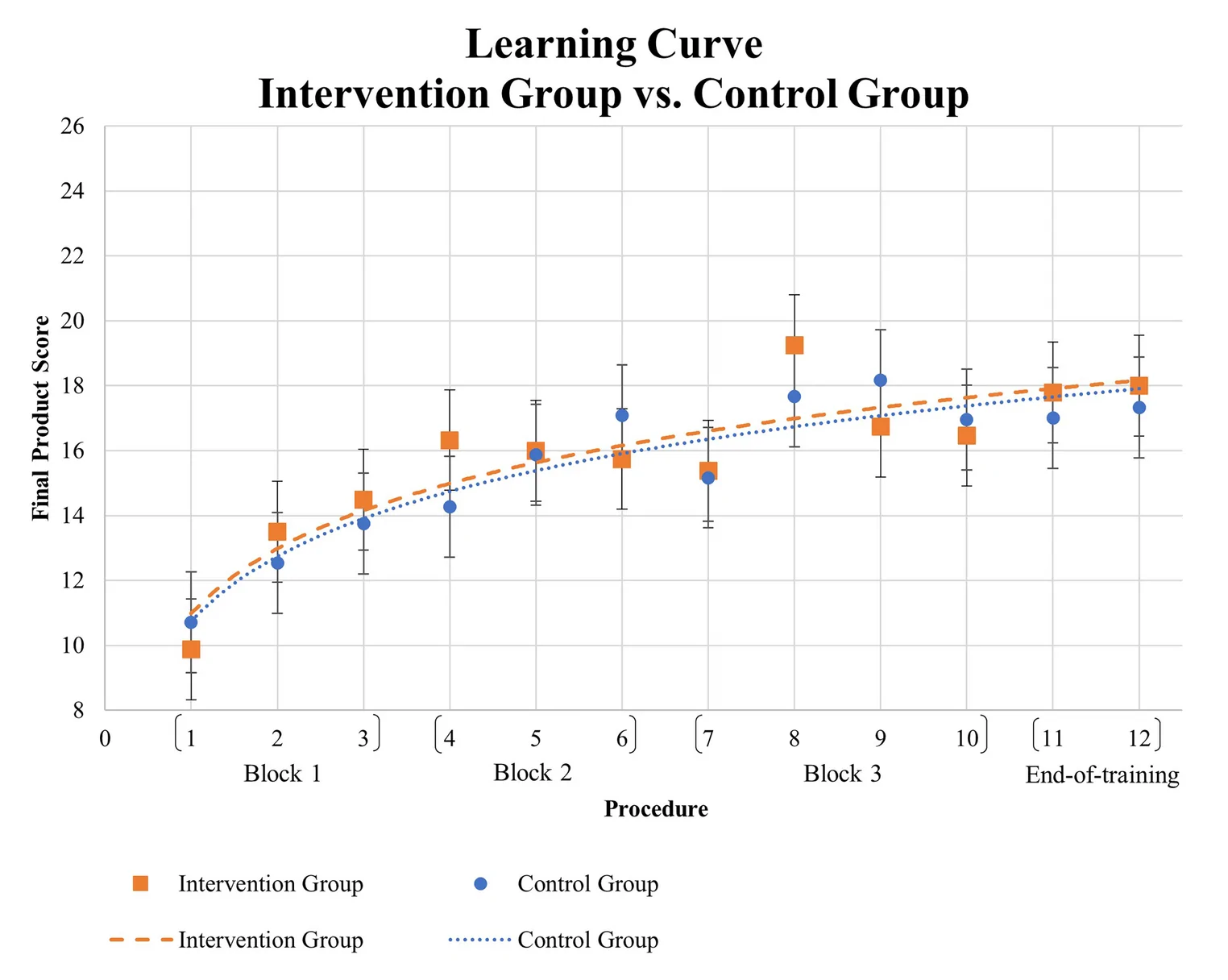
Marginal mean values with CI 95% for the final product for both groups, with a fitted curve, representing the learning curve. Maximum score: 26 points (y-axis)

The learning curves, demonstrating the relationship between mean performance scores and number of procedures, were plotted for each group (Fig. 3). Performance improved with increasing repetition number across both groups (LMM; *P* < 0.001); however, the rate of improvement did not differ between the intervention and control group (LMM; *P* = 0.53). The average learning curves from the two groups demonstrates a steep improvement from the first to the second training block (performance 1–3) after which the performance gradually starts to plateau. The baseline scores for the intervention group and the control group were 9.9 points; 95% CI [8.5–11.4] and 10.7 points; 95% CI [9.1–12.3] (*p* = 0.46), respectively, out of a maximum of 26 points. There was no significant difference in improvement between the intervention and control group from baseline to end-of-training (*p* = 0.52): at end-of-training, the intervention group had improved by 7.9 points (95% CI [5.7–10.2], *p* < 0.001), corresponding to a 80% score improvement and the control group by 6.3 points (95% CI [4.2–8.4], *p* < 0.001), equaling a 59% score improvement.

### Cognitive load

Cognitive load was similar in the two groups. During the first procedure, the intervention group had a relative reaction time of 1.49 (95% CI [1.44–1.53] vs. 1.47 (95% CI [1.43–1.51] in the control group, i.e. reaction time increased 49% for the intervention group and 47% for the control group compared to baseline measurements.

**Fig. 4:**
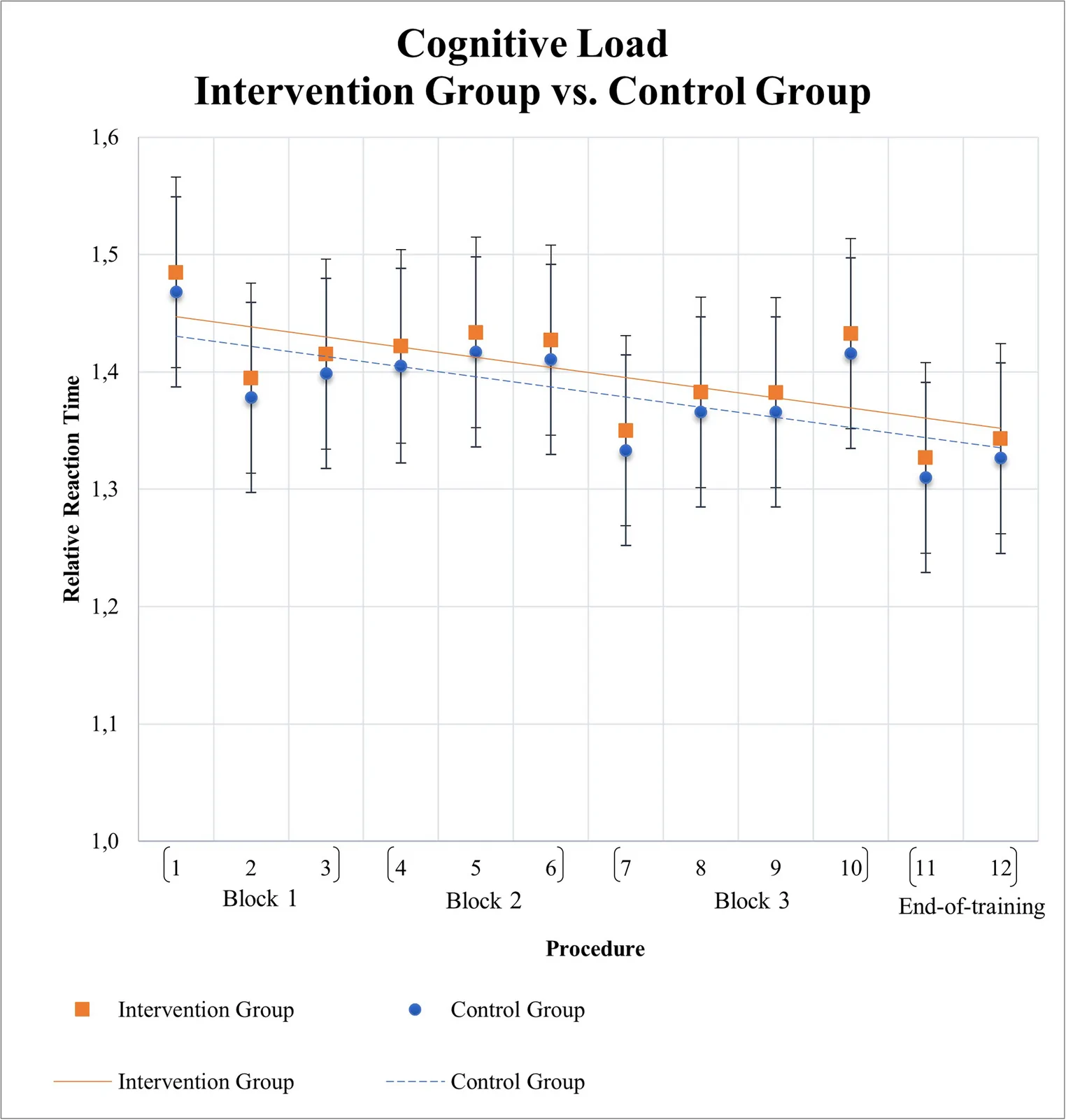
CL based on the secondary task relative reaction time. Intervention group compared to Control group with 95% confidence intervals (vertical bars) and linear regression

At end-of-training, the relative reaction time was 1.33 (CI 95% [1.29–1.37] vs. 1.31 (CI 95% [1.27–1.35], for the intervention -and control groups respectively. This corresponds to a decrease of 11.6% (0.17 CI 95% [0.083–0.247], *p* < 0.001) and 10% (0.15 CI 95% [0.07–0.23), *p* < 0.001; Fig. 4), respectively, compared to baseline.

## Discussion

In this randomized controlled educational trial, we investigated the effects of AV on surgical skills acquisition and CL during VR simulation-based mastoidectomy training. Contrary to our initial hypothesis, we found no significant differences between the intervention or control groups regarding performance or CL. Both groups exhibited comparable learning curves, plateauing at 68% and 65% of the maximum score, respectively. These results indicate that participants in both groups were still unable to safely perform several crucial steps of the procedure even at the end-of-training. In both groups, considerable CL was induced, but decreased with increasing amount of training.

The lack of effects from AV training suggests that the introduction of AV did not substantially impede initial skills acquisition, nor did it elevate performance before plateauing. Also, as relative reaction time was similar in the two groups throughout training, the intervention did not seem to affect CL and the formation of mental schemata. This could be caused by participants not having any spare attentional resources to focus on AV, possibly due to lack of knowledge of the complex anatomy, making the AV too subtle for the participants to notice. The novices were both trained and tested only on anatomies ranging within normal anatomical limits. Moreover, although performances improved, the learning curves never reached the level required for clinical competency.

Although anatomical variation has been suggested as a beneficial instructional design feature, few studies have investigated it, particularly in relation to surgical performance [5, 32]. Previous studies investigating use of anatomical variations in simulation training found similar results to our study, with little effect on overall performance [33, 34]. Specific for the mastoidectomy procedure, a recent study investigated whether training on case-specific 3D-printed models resulted in better subsequent cadaver dissection for novices compared to training on a generic model. Also here, AV did not result in better performance [35]. Another randomized trial, featuring five and six participants in each group, evaluated AV in VR mastoidectomy training assessing end-of-training performances, and found no significant effect of AV on overall performance [8]. This generally aligns with our findings, but in contrast to the previous studies, we evaluated the entire learning curve and included data on CL, to elucidate not only *whether* but also *why* the intervention did or did not work.

The concept of *desirable difficulties*, described by Bjork, entails creating a level of difficulty of a task that enables consolidation and retrieval of skills, through supporting learning, understanding and recollection [6]. A difficulty must match the level of competency of the learner, otherwise it becomes an *undesirable difficulty*, which will not aid learning. We suspect that the type of anatomical variations introduced in this trial did not match the participating novices’ low level of anatomical knowledge of the temporal bone. This would make it difficult for participants to adjust their decision making relevantly, thus not becoming a desirable difficulty. Correspondingly, AV might have more impact on learning and performance at a later stage in surgical training than with novices, i.e., in residents who are familiar with the basic steps and anatomical details of the procedure.

The strengths of our study include the randomized design, blinded assessment by two expert raters and use of learning curves, i.e., longitudinal assessment of skills acquisition encompassing 12 performances. This is, to our knowledge, one of the biggest studies of AV in surgical training when it comes to number of assessed performances. Further, we used objectively measured estimates of CL to elucidate the effect on the intervention on a cognitive level.

Our findings are limited by the study population of novices who likely lack sufficient anatomical understanding to act on the natural variations of a normal patient population. Correspondingly, the moderate performance does not comprehensively incorporate all elements required to master the procedure to a level required during clinical surgery. Furthermore, the use of medical students without prior surgical experience limits the external validity of our findings to more experienced learners such as residents. We chose medical students as novice trainees as this approach is well-established in the mastoidectomy training literature and minimizes the confounding effect of prior clinical exposure allowing the effect of AV to be tested under experimental control. The present study was designed to test AV in VR training under such controlled conditions, and the findings are intended to inform the design of future training curricula rather than to directly simulate residency training. Consequently, if the effect of AV differs according to the level of training remains unknown.

For creating a training curriculum targeted novice mastoidectomy learners, future studies should investigate other instructional designs that could potentially match desirable difficulties. Key for novice learners is to overcome the current learning plateau, that is reached after only approximately nine VR procedures, fast and effectively. Such instructional design could be timely progression to an additional modality, such as 3D-printed models. Combining two effective simulation modalities (i.e. 3D-printed models and VR training) could have an additive effect on mastoidectomy performance but remains unexplored. A comprehensive understanding is required for creating a robust and effective curriculum ensuring trainee competency before supervised surgery.

## Conclusion

This randomized controlled trial does not support the hypothesis that anatomical variation significantly impacts skills acquisition, i.e., the learning curve, or cognitive load during virtual reality SBT of mastoidectomy in complete novices. Thus, other elements of the training framework should be prioritized when it comes to learning basic surgical skills at a novice level.

The use of AV could still prove to be a valuable element in simulation training, for instance in other surgical procedures or more experienced trainees.

## Data Availability

Available on reasonable request.
